# In vivo evidence for the contribution of peripheral circulating inflammatory exosomes to neuroinflammation

**DOI:** 10.1186/s12974-017-1038-8

**Published:** 2018-01-08

**Authors:** Jing Jing Li, Bin Wang, Mahesh Chandra Kodali, Chao Chen, Eunhee Kim, Benjamin John Patters, Lubin Lan, Santosh Kumar, Xinjun Wang, Junming Yue, Francesca-Fang Liao

**Affiliations:** 1 Department of Pharmacology, University of Tennessee Health Science Center, 71 South Manassas Street, Memphis, TN 38103 USA; 2 Department of Neurosurgery, The Fifth Affiliated Hospital of Zhengzhou University, Zhengzhou, China; 3 Department of Pharmaceutical Science, University of Tennessee Health Science Center, Memphis, USA; 4 Department of Pathology and Laboratory Medicine, University of Tennessee Health Science Center, Memphis, USA

**Keywords:** Neuroinflammation, Lipopolysacchride, Microglia, Exosomes

## Abstract

**Background:**

Neuroinflammation is implicated in the development and progression of many neurodegenerative diseases. Conditions that lead to a peripheral immune response are often associated with inflammation in the central nervous system (CNS), suggesting a communication between the peripheral immune system and the neuroimmune system. The underlying mechanism of this relationship remains largely unknown; however, experimental studies have demonstrated that exposure to infectious stimuli, such as lipopolysaccharide (LPS) or high-fat diet (HFD) feeding, result in profound peripheral- and neuro-inflammation.

**Methods:**

Using the model of endotoxemia with LPS, we studied the role of serum-derived exosomes in mediating neuroinflammation. We purified circulating exosomes from the sera of LPS-challenged mice, which were then intravenously injected into normal adult mice.

**Results:**

We found that the recipient mice that received serum-derived exosomes from LPS-challenged mice exhibited elevated microglial activation. Moreover, we observed astrogliosis, increased systemic pro-inflammatory cytokine production, and elevated CNS expression of pro-inflammatory cytokine mRNA and the inflammation-associated microRNA (miR-155) in these recipient mice. Gene expression analysis confirmed that many inflammatory microRNAs were significantly upregulated in the purified exosomes under LPS-challenged conditions. We observed accumulated signaling within the microglia of mice that received tail-vein injections of fluorescently labeled exosomes though the percentage of those microglial cells was found low. Finally, purified LPS-stimulated exosomes from blood when infused directly into the cerebral ventricles provoked significant microgliosis and, to a lesser extent, astrogliosis.

**Conclusions:**

The experimental results suggest that circulating exosomes may act as a neuroinflammatory mediator in systemic inflammation.

**Electronic supplementary material:**

The online version of this article (10.1186/s12974-017-1038-8) contains supplementary material, which is available to authorized users.

## Background

Neuroinflammation is a common pathological feature of neurodegenerative diseases such as Alzheimer’s disease (AD), Parkinson’s disease (PD), frontotemporal lobar dementia (FTD), and amyotrophic lateral sclerosis (ALS) and is represented by glial activation and pro-inflammatory cytokine production by the central nervous system (CNS)-resident cells [1]. Epidemiological studies have revealed that therapeutic use of nonsteroidal anti-inflammatory drugs (NSAIDs) in humans reduces the risk of developing AD and PD [2–4], suggesting that components of the inflammatory pathways may be involved in the pathogenesis of neurodegenerative diseases. Clinical trials designed to slow AD progression by targeting these inflammatory pathways have failed to provide evidence of efficacy [5]; however, experimental findings from rodents and genome-wide association studies (GWAS) suggest a direct association between neuroinflammation and the development and progression of neurodegeneration [6–12].

Neuroinflammation is a phenomenon that frequently occurs with systemic inflammatory conditions, such as rheumatoid arthritis [13], sepsis [14], type 2 diabetes [15], and obesity [16]. This suggests a link between the peripheral immune system and the neuroimmune system; however, the underlying mechanisms of this crosstalk remain largely unknown. Previous experimental studies have shown that peripheral administration of lipopolysaccharide (LPS), the major outer membrane component of gram-negative bacteria, or high-fat diet (HFD) feeding induces a profound innate immune response not only in peripheral organs but also in CNS in rodents [17–22]. Exosomes are a group of cell-derived vesicles that are 30–100 nm in size, produced by most cells in the body, and capable of carrying various cargo molecules including mRNA, microRNA, lipids, and proteins. When these vesicles are released into the circulatory system, they are transported to distal sites and internalized into target cells through endocytosis or membrane fusion [23, 24]. Exosomes are the new mediators in cell-to-cell communication between neighboring cells or distant cells. We hypothesize that the blood-borne exosomes may be involved in the crosstalk between peripheral and CNS immune system.

## Methods

### Animals

C57BL/6J mice were obtained from Jackson Laboratories. Mice were housed and bred in the animal care facility at the University of Tennessee Health Science Center under a 12/12 h light/dark cycle with ad libitum access to food and had similar body weights unless otherwise specified.

### LPS injection

In the LPS model, mice of age 3–4 months were injected intraperitoneally (i.p.) with either LPS (*Escherichia coli* 055:B5, Sigma-Aldrich, St Louis, MO, USA) or phosphate-buffered saline (PBS); in some experiment, we used saline as control. In previous studies of CNS inflammatory response using the LPS model, an i.p. injected dose of LPS between 0.5–10 mg/kg was generally used to induce a whole body immune response, which included neuroinflammation. In the current study, to study the effects of the temporal- and dose-dependent changes on CNS inflammation, we implemented additional treatment regimens: (1) mice were euthanized 1, 6, and 24 h after a low dose i.p. injection of LPS at 0.5 mg/kg; (2) mice were euthanized after 7 days of daily low dose i.p. injection of LPS at 0.5 mg/kg; (3) mice were euthanized 1 and 6 h after a high dose i.p. injection of LPS at 10 mg/kg. Mice receiving low-dose LPS (0.5 mg/kg) did not show any behavioral changes, while the mice receiving high-dose LPS (10 mg/kg) became severely ill and immobile 3–4 h following injection. To rule out overnight dehydration as a confounding variable, we removed the 24-h time-point in the 10 mg/kg regimen group from our study design. Donor mice receiving i.p. injection of LPS at 5 mg/kg for 24 h were specified in corresponding experiments. Equal numbers of male and female mice were included.

### Exosome isolation, quantification, size and zeta potential measurement, and labeling

Exosomes were isolated from the sera by the ExoQuick serum exosome precipitation solution according to the manufacturer’s instructions (EXOQ5A-1, Systems Biosciences, San Francisco, CA, USA). For differential ultracentrifugation as used in Fig. 2e–g, the serum samples were centrifuged at 20,000×*g* at 4 °C for 30 min to remove debris and were then centrifuged at 100,000×*g* at 4 °C for 2 h using Sw50.2Ti rotor. The pellets which should be enriched for exosomes were resuspended in 1× PBS. The suspension was centrifuged again at 100,000×*g* at 4 °C for 2 h. We purified exosomes from kit unless stated otherwise. For exosome protein quantification, the exosomes were resuspended in PBS (1/5 volume of the input serum) and a portion of the suspension was mixed with 2× radioimmunoprecipitation (RIPA) buffer. The lysates were centrifuged at 12000×*g* at 4 °C for 10 min. The supernatant was subsequently quantified by Pierce™ BCA protein assay (23225, Thermo Fisher Scientific, Waltham, MA, USA). For exosome size and zeta potential measurement, the exosome pellets were resuspended in DNase/RNase-free water; size and zeta potential determination of isolated exosomes was performed using Zetasizer Nano-Z (Malvern Instruments, Worcestershire, UK). Exosome labeling was performed using an Exo-Glow Exosome Cargo Labeling Kit according to the manufacturer’s instructions (EXOG200A-1, Systems Biosciences, San Francisco, CA, USA).

### Administration of LPS-stimulated blood exosomes from donor to recipient mice

Whole blood (700–800 μl) from mice treated either with or without LPS was collected by cardiac puncture. Blood was centrifuged at 2000×*g* for 10 min after sitting undisturbed at room temperature for 30 min to separate the serum. Purified exosomes from the sera were then resuspended in 200 μl of sterilized 1× PBS and passed through a 0.22-μm filter before tail-vein injection to the recipient mice. In our pilot study, we transfused three different doses (500 μg, 1 mg, and 1.5 mg) of exosomes from mice treated with high-dose (10 mg/kg, 6 h) LPS and found that both 1 and 1.5 mg of exosomes induced significant increase of microglia activation in the recipient mice while 500 μg failed to achieve such effect. We therefore used a 1 mg exosome dose throughout the remainder of the study. For the follow-up experiments of infusing LPS-exosomes to recipient mice via the intracerebroventricular (i.c.v.) route, whole blood was collected from the donor mice and the exosomes were isolated from sera using ExoQuick kit and resuspended in sterile PBS (1 mg in 10 μl of saline). Intraventricular infusion of 1 mg of exosomes in 10 μl was performed via implanted cannula, as described in our previous work [25], over a course of 1 h at the speed of 0.16 μl/min. Mice were all perfused 24 h after receiving exosomes followed up by immunohistochemistry examination of inflammatory markers.

### Immunofluorescent staining

After perfusion, mouse brains were dissected and fixed in 4% paraformaldehyde/PBS for 24 h followed by a cryopreservation step in 30% sucrose/PBS for 3 days. Brains were embedded in optimal cutting temperature (OCT) compound (#23-730-571, Fisher Scientific, Hampton, NH, USA) and sectioned coronally on a Leica CM1850 cryostat at a thickness of 20 μm. Sections were attached to microscope slides (#12-550-15, Fisher Scientific, Hampton, NH, USA) and allowed to dry on a slide warmer for an hour at 37 °C. Tissue sections were permeabilized with 0.1% Triton X-100/PBS for 10 min at room temperature. Non-specific bindings were blocked using 5% goat serum/PBS for an hour at room temperature. Primary antibodies diluted in antibody dilution buffer (#25886-05, Electron Microscopy Sciences, Hatfield, PA, USA) were applied to slides for overnight incubation at 4 °C. Antibodies used in this study include: rabbit anti-Iba-1 (#019-19741, Wako Laboratory Chemicals, Richmond, VA, USA; 1:1000), mouse anti-GFAP (G3893, Sigma-Aldrich, St Louis, MO, USA; 1:500), rat anti-CD68 (MCA1957, Bio-Rad Laboratories, Hercules, CA, USA; 1:200), and rabbit anti-S100 (Ab41548, Abcam, Cambridge, MA; 1:1000). On the second day, slides were washed for 5 min × 3 times with PBS at room temperature, followed by the incubation of an Alexa Fluor secondary antibody (Thermo Fisher Scientific, Waltham, MA, USA; 1:200) for an hour at room temperature. After three washes, the sections were counterstained with DAPI, air dried, and mounted with Fluoromount-G (# 0100-01, SouthernBiotech, Birmingham, AL, USA). Our pilot study revealed that the Exo-Green dye faded quickly after use, therefore the brains were rapidly isolated, snap frozen with crushed dry ice. The fresh frozen brains were embedded in OCT compound and sectioned coronally at 20 μm. Sections were mounted on microscope slides and briefly fixed in 4% paraformaldehyde/PBS for 15 min followed by three PBS washes. After tissue permeabilization and blocking, the sections were incubated with primary antibody overnight at 4 °C.

### Digital image quantification

Images with a resolution of 4080 × 3072 pixels were captured with an Olympus IX50 microscope (Olympus Corporation, Shinjuku, Tokyo, Japan). Images were acquired within the hippocampal and neocortical regions. A total of ~ 50 coronal sections (20 μm per section) through the hippocampal region of one animal brain were cut, and one section from every eight sections were collected to constitute a six-section serial set from which we quantified the staining intensity. Before image acquisition, we generally looked through each section to see if the hippocampal and neocortical structures were intact and if the section was flattened but not folded up. Six to eight images per structure per animal were captured, covering as many sections as possible from the six-section serial set. Exposure time was manually adjusted to minimize saturation while maintaining adequate signal-to-background contrast. Images from the same antibodies were acquired using the same exposure time. The images were converted to 680 × 512 pixels and analyzed using ImageJ software (National Institutes of Health, Bethesda, MD; http://imagej.nih.gov/ij/). Image files were converted to RGB stacks and color-inverted, converting the color images to grayscale mode. Then, we adjusted the threshold of the images to define the positive signals from the surrounding background. The total staining intensity were expressed by integrated intensity (i.e., mean gray value × area) using the ROI manager function of ImageJ. Areas of positive aggregates were identified between 3 to 500 pixel^2 with circularity of 0 to 1. Areas of false-positive aggregates with no cell morphology were manually excluded. The average value from the six to eight images was used to represent the value from one mouse brain. The number of CD68+ cells was counted manually.

### Quantitative real-time polymerase chain reaction (qRT-PCR)

Either 100 μl of whole blood, 100 mg of liver, or half forebrain was used as input. Total RNA was isolated using Trizol reagent (Invitrogen, Carlsbad, CA, USA). For mRNA analysis, the cDNAs were synthesized using High-Capacity cDNA Reverse Transcription Kit (4368814, Thermo Fisher Scientific, Waltham, MA, USA). For brain microRNA analysis, the cDNAs were synthesized by miScript II RT Kit (218,161, Qiagen, Germantown, MD, USA). For exosomal microRNA analysis, the cDNAs were synthesized by Complete SeraMir Exosome RNA Amplification kit (RA800A-1, Systems Biosciences, San Francisco, CA, USA) according to the manufacture’s protocol. An equal volume of input serum was used for the same batch of samples. The expression of microRNA was normalized to a synthetic external spike-in small RNA control (RA805A-1, Systems Biosciences, San Francisco, CA, USA). Detection of mRNA or microRNA were performed using with SsoAdvanced™ Universal SYBR® Green Supermix (Bio-Rad Laboratories, Hercules, CA, USA) and an Eppendorf Mastercycler realplex Real-Time PCR system. Primers used were the following: *Tnf*, forward 5′-CCCTCACACTCAGATCATCTTCT-3′, reverse 5′-GCTACGACGTGGGCTACAG-3′; *Il6*, forward 5′-AGTTGCCTTCTTGGGACTGA-3′, reverse 5′-TCCACGATTTCCCAGAGAAC-3′. Their expressions were normalized to β-actin: *Actb*, forward 5′-CTAAGGCCAACCGTGAAAAG-3′, reverse 5′-ACCAGAGGCATACAGGGACA-3′. Primers for mature microRNAs were designed using the mouse sequence from miRBase. Their expressions were normalized either to the spike-in small RNA control in exosomal microRNA analysis, or to 5S rRNA: 5S, forward 5′-GCCCGATCTCGTCTGATCT-3′; reverse 5′-GCCTACAGCACCCGGTATC-3′ in brain microRNA analysis.

### Western blot analysis

Around 20 μg of protein per sample was used for western blot analyses performed as described previously [26]. Antibodies used includes anti-TSG101 antibody (SC-7964, Santa Cruz Biotechnology, Dallas, TX, USA) and anti-β-Actin antibody (#A2228, Sigma-Aldrich, St Louis, MO, USA).

### Enzyme-linked immunosorbent assay (ELISA)

TNF-α levels were measured using Mouse TNF-α Quantikine ELISA Kit according to the manufacturer’s instructions (MTA00B, R&D Systems, Minneapolis, MN, USA).

### HFD feeding

In the diet-induced obesity model, 6-week-old male mice were fed either a HFD containing 60% calories from fat (TD. 06414, Harlan Teklad, Indianapolis, IN, USA) or a standard chow diet for 16 weeks. Mouse body weights were measured before and after the 16 weeks of diet feeding.

### Statistics

A Kolmogorov–Smirnov test was used to determine if the values were normally distributed. Statistical analysis was performed using one-way ANOVA analysis followed by a Bonferroni post hoc test for multiple comparisons. Student’s *t* test was performed when comparing two means. *P* values of < 0.05 were considered significant. Graphs were analyzed and prepared using GraphPad Prism 5.0 (La Jolla, CA, USA).

## Results

### Rapid microglial and astrocytic activation in the CNS by intraperitoneally administered LPS

**Fig. 1:**
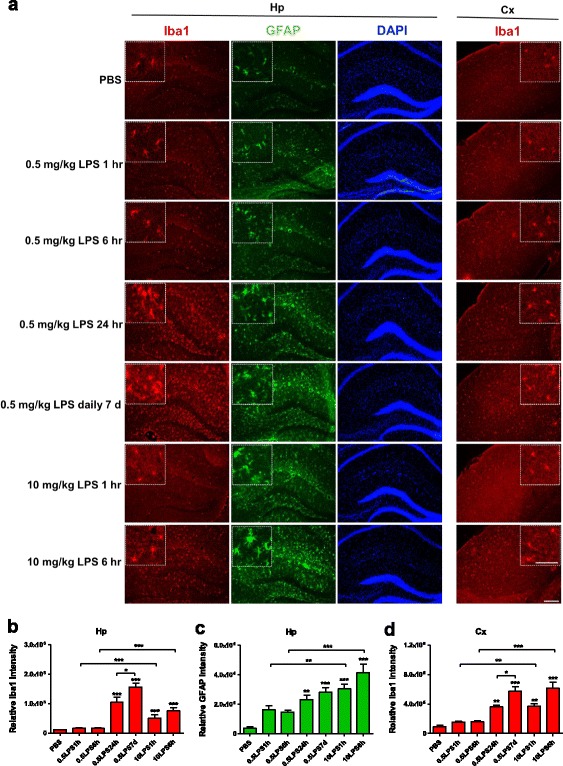
Increased microglial and astrocytic activation after intraperitoneally injected LPS. **a** Photomicrographs of the hippocampal (Hp) and neocortical (Cx) areas from mice intraperitoneally injected with either PBS or treated with one of the three LPS regimens. Brain sections were stained with anti-Iba1 antibody for microglia (red) or with anti-GFAP antibody for astrocytes (green). DAPI was shown in blue. Scale bar, 50 μm. The white-dotted boxes are zoom-in views of the corresponding photomicrographs, scale bar 10 μm. **b**, **c** Comparison of the relative immunofluorescent intensity of Iba1 (**b**) and GFAP (**c**) in the hippocampal area between different treatments. **d** Comparison of the relative immunofluorescent intensity of Iba1 in the neocortical area between treatments. *n =* 5–8 per group. Data represent mean ± SEM; **P < 0.01 and ***P < 0.001

In the low-dose LPS (0.5 mg/kg) treatment group, the microglial response (the main outcome parameter of CNS inflammation) peaked at 24 h post-injection in both hippocampal and neocortical areas. Multiple injections for 7 days further increased the microglial response. Of the mice treated with 10 mg/kg LPS, the microglial response was increased more dramatically at 1 and 6 h when compared with the same time-points in the 0.5 mg/kg LPS treatment group (Fig. 1a–d). Peaked astrocytic response in the hippocampus was also observed at 24 h post-injection in low-dose LPS regimen group. Multiple injections for 7 days failed to further increase the GFAP signal intensities, while high-dose LPS regimen further increased GFAP signal intensities at 1 and 6 h when compared with the low-dose regimen (Fig. 1a, c). The number of Iba1+ and GFAP+ cells was also quantified which shows similar degrees of increase in response to different LPS administration regimens (Additional file 1: Figure S1).

### Intravenously administered serum-derived exosomes purified from LPS-challenged mice induce microglial and astrocytic activation in the CNS

**Fig. 2:**
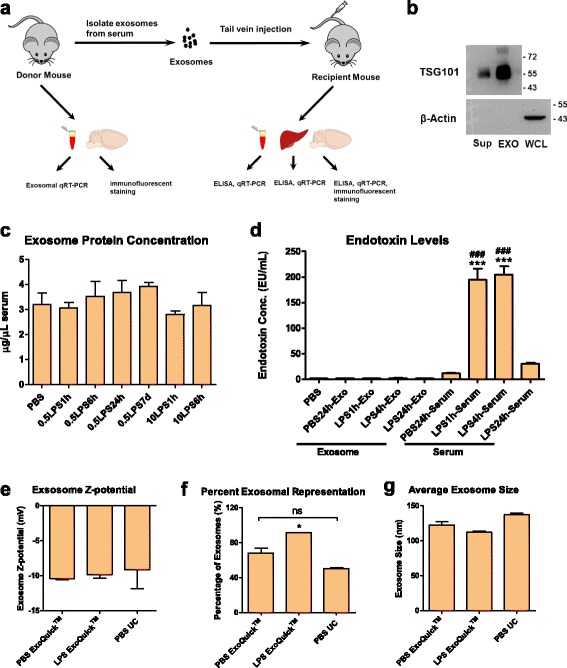
Characterization of purified exosomes. **a** A schematic diagram of the experimental design. Exosomes isolated from sera of the donor mice were injected to the recipient mice via tail-vein. Serum and brain of the donor mice were used for exosomal inflammatory microRNA detection and immunofluorescent staining of microglia/astrocyte marker, respectively. Whole blood, serum, liver, and brain were collected from the recipient mice 24 h after injection. Whole blood, liver, and brain tissues were used for examining the mRNA expression of pro-inflammatory cytokines (such as TNF-α and IL-6) by qRT-PCR. TNF-α concentrations were measured by ELISA in serum, liver and brain homogenates. **b** Representative western blot image showing enriched TSG101 expression in the exosomal preparation. Sup supernatant, EXO exosomal preparation, WCL whole cell lysate. **c** Protein concentrations of exosomes in the sera from mice treated with LPS were not altered. *n* = 3–5 per group. **d** Endotoxin levels in exosome suspension or serum from PBS- or LPS-treated mice. Sera were collected from mice 1, 4, or 24 h after LPS i.p. injection. *n* = 3–4 per group. **e** Zeta potential of exosomes purified from the sera of mice treated for 6 h with PBS or 10 mg/kg LPS either by ExoQuick kit (ExoQuick™) or by differential ultracentrifugation (UC), *n* = 3 per group. **f** Percentage of exosomes in the size range of 10–200 nm. **g** Average exosome size. Data represent mean ± SEM; ns no statistical significance, *P < 0.05, ***P < 0.001, ^###^*P* < 0.001 as compared to the PBS24h-serum group

To investigate whether the serum-derived exosomes mediate LPS-induced neuroinflammation, we purified exosomes from the sera of the LPS-challenged donor mice and transfused them to the recipient C57BL/6J young adult mice via tail-vein injection (Fig. 2a). We first purified serum-derived exosomes from mice from the three LPS treatment regimen groups using ExoQuick kit. Western blot analysis revealed that the exosome marker protein TSG101 was markedly enriched in these exosomal preparation, in which the cellular marker β-Actin was not detected, suggesting that we have bona-fide exosomes in our study (Fig. 2b). The protein concentrations in these exosomes were unchanged as measured by a BCA protein assay, suggesting that LPS-induced systemic inflammation may not alter exosome secretion profile in the sera (Fig. 2c). Endotoxin test results indicated that these exosomes isolated from mice at 1, 4, or 24 h after LPS injection had barely detectable endotoxin contamination, whereas the sera from these mice contained 194.80 ± 18.53 endotoxin units/ml (EU/ml) and 204.61 ± 12.47 EU/ml at 1 and 4 h after LPS injection, respectively. The endotoxin level in sera dropped to 29.98 ± 1.67 EU/ml at 24 h after the injection (Fig. 2d). We also measured the size and Zeta potential of exosomes purified by ExoQuick kit and compared those with exosomes purified by the classic differential ultracentrifugation method. We found that both methods yielded exosomes with Zeta potential around − 10 mV, which was within the ideal range of − 8~− 12 mV, suggesting that the ExoQuick method did not alter the property of exosomes as compared with the ultracentrifugation method (Fig. 2e). LPS treatment did not cause any change in Zeta potential; however, treatment of high-dose LPS (10 mg/kg for 6 h) slightly but significantly increased the percentage of exosomes that are between 10 and 200 nm (Fig. 2f). The average size of exosomes was not significantly varied between groups (Fig. 2g). These results indicate that ExoQuick kit is sufficient, as well as a simple and cost-effective method to purify exosomes from mouse sera. The subsequent studies were all performed using the exosomes purified by this method.

**Fig. 3:**
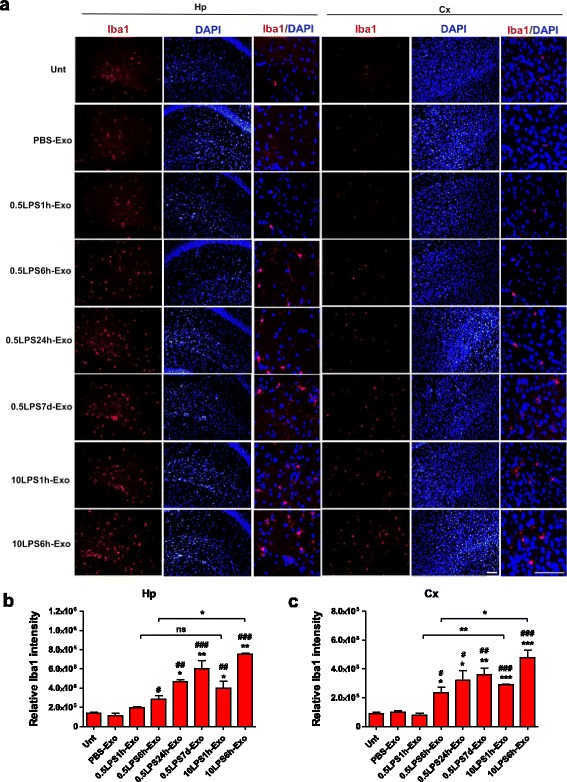
Exosomes isolated from LPS-treated mice increase microglial activation. **a** Photomicrographs of the hippocampal (Hp) and neocortical (Cx) areas from C57BL/6J mice. Mice were either left untreated (Unt) or intravenously injected 1 mg of exosomes (in 200 μl PBS) isolated from the sera of donor mice. Donors received intraperitoneal injection of PBS or treatment of one of the three LPS regimens. The recipient mice were euthanized after 24 h. Brain sections were stained with anti-Iba1 antibody for microglia (red) and with DAPI for nuclei (blue). Scale bar, 20 μm. **b**, **c** Comparison of the relative immunofluorescent intensity of Iba1 in the hippocampal (**b**) and neocortical (**c**) areas between treatments. *n* = 3–5 per group. Data represent mean ± SEM; ns no statistical significance, *P < 0.05, **P < 0.01, and ***P < 0.001; ^#^*P* < 0.05, ^##^*P* < 0.01, and ^###^*P* < 0.001 as compared to the PBS-Exo-treated group

**Fig. 4:**
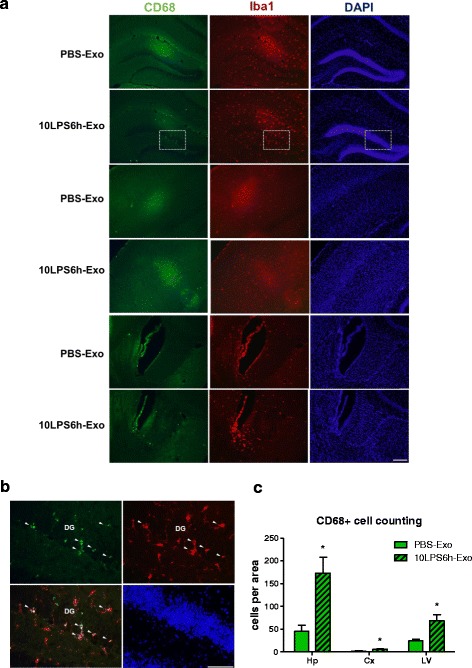
Exosomes isolated from LPS-treated mice increase CD68-positive cells. **a** Photomicrographs of the hippocampal (Hp), neocortical (Cx), and lateral ventricular (LV) areas from C57BL/6J mice. Mice were intravenously injected 1 mg of exosomes (in 200 μl PBS) isolated from the sera of donor mice 6 h after intraperitoneal injection of PBS or 10 mg/kg LPS. The recipient mice were euthanized after 24 h. Brain sections were stained with anti-CD68 antibody (green) and DAPI (*blue*). Scale bar, 50 μm. **b** Higher magnification images of the white-dotted box area from **a**. Figures are presented in the following sequence: upper left, CD68 (green); upper right, Iba1 (red); lower left, merge of CD68 and Iba1; and lower right, DAPI (blue). Co-localization of CD68 and Iba1 (white arrowheads). DG dentate gyrus. Scale bar, 20 μm. **c** Quantification of CD68-positive cells in the hippocampal, neocortical, and lateral ventricular areas. *n* = 3–5 per group. Data represent mean ± SEM; *P < 0.05

**Fig. 5:**
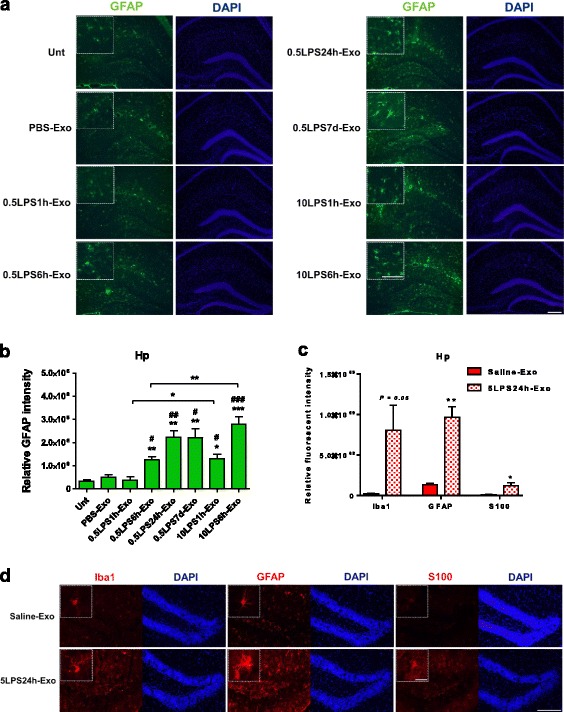
Exosomes isolated from LPS-treated mice increase astrocyte activation. **a** Photomicrographs of the hippocampal area from C57BL/6J mice. Mice were either left untreated (Unt) or intravenously injected 1 mg of exosomes (in 200 μl PBS) isolated from the sera of donor mice. Donors were intraperitoneally injected with PBS or treated with one of the three LPS regimens. The recipient mice were euthanized after 24 h. Brain sections were stained with anti-GFAP antibody for astrocytes (green) and with DAPI for nuclei (blue). Scale bar, 50 μm. The white-dotted boxes are zoom-in views of the corresponding photomicrographs, scale bar 10 μm. **b** Comparison of the relative immunofluorescent intensity of GFAP in the hippocampal area between treatments. *n* = 3–5 per group. **c** Quantification of the relative immunofluorescent intensity of Iba1, GFAP, and S100 in the hippocampal area in Saline-Exo- and 5LPS24h-Exo-treated mouse brains. The donor mice received either saline or 5 mg/kg of LPS injection and were kept alive for 24 h before blood collection. The recipient mice were i.v. injected with the purified exosomes and kept alive for 24 h before being sacrificed. *n* = 3 per group. **d** Representative photomicrographs of the immunofluorescent staining in the brain sections from Saline-Exo- and 5LPS24h-Exo-treated mice. Anti-Iba1 antibody for microglia, anti-GFAP and anti-S100 antibodies for astrocytes (red), and DAPI for nuclei (blue). Scale bar, 50 μm. The white-dotted boxes are zoom-in views of the corresponding photomicrographs, scale bar 5 μm. Data represent mean ± SEM; *P < 0.05, **P < 0.01, and ***P < 0.001; ^#^*P* < 0.05, ^##^*P* < 0.01, and ^###^*P* < 0.001 as compared to the PBS-Exo-treated group

We then determined neuroinflammation induced by tail-vein-injected exosomes. Histological staining for Iba1 in the recipient mice was performed to examine the microglial response in the hippocampal and neocortical areas. Exosomes purified from mice treated with 0.5 mg/kg LPS for 6 h (0.5LPS6h-Exo) and for 24 h (0.5LPS24h-Exo), but not those treated with 0.5 mg/kg LPS for 1 h (0.5LPS1h-Exo) or PBS (PBS-Exo) significantly increased microglia activation in the recipient mice after 24 h in comparison with the untreated mice, suggesting that the 0.5LPS6h-Exo and 0.5LPS24h-Exo are more inflammatory than the 0.5LPS1h-Exo and PBS-Exo. Consistent with the effects of LPS treatment alone, 10LPS1h-Exo and 10LPS6h-Exo induced a greater CNS inflammatory response than did 0.5LPS1h-Exo and 0.5LPS6h-Exo. Multiple LPS injections resulted in exosomes with similar inflammability when compared with single LPS injections (Fig. 3). To verify that microglia activation was increased by exosomes isolated from LPS-treated mice, we stained the brain slices with anti-CD68 antibody to identify activated microglia. The number of CD68-positive cells was counted manually in the hippocampal, neocortical, and lateral ventricular areas in mice treated with PBS-Exo or 10LPS6h-Exo. Our results showed that 10LPS6h-Exo increased the number of activated microglial cells in all three brain regions to varying degrees (Fig. 4). Similar observations were made in the astrocytic response in the hippocampal regions, as indicated by the GFAP fluorescent intensity (Fig. 5a, b). Staining the brain slices of LPS-Exo-treated mice for an additional astrocyte marker S100 confirmed the activation of astrocytes (Fig. 5c, d, and Additional file 2: Figure S2).

### Intravenously administered serum-derived exosomes purified from HFD-fed mice induce microglial activation in the CNS

HFD-induced obesity is associated with chronic low-grade peripheral inflammation [27, 28] and is also associated with noticeable CNS inflammation [18, 29–32]. Consistent with reported studies, we found that HFD-fed mice displayed higher levels of Iba1 intensity in hippocampal and cortical regions when compared with the Chow-fed mice (Additional file 3: Figure S3a-c). After 16 weeks of diet feeding, HFD-fed mice gained significantly more weight than the Chow-fed mice (Additional file 3: Figure S3d). It remains unclear whether it was in fact the HFD or its dietary metabolites that directly caused neuroinflammation or perhaps it was the molecules produced during the development of obesity that was responsible for mediating the inflammatory effects in the CNS. To test the hypothesis that serum-derived exosomes may act as a mediator in obesity-associated neuroinflammation, we transfused serum exosomes isolated from mice fed with a chow diet or a HFD for 16 weeks to the recipient C57BL/6J young adult mice and waited 24 h before sacrificing the recipient mice for histological analysis. Our results showed that exosomes isolated from HFD-fed mice (HFD-Exo) induced approximately twofold increase in Iba1 fluorescent intensity in hippocampus and neocortex of recipient mice over Chow-Exo (Additional file 3: Figure S3e-g).

### Intravenously administered serum-derived exosomes purified from LPS-challenged mice increase systemic and CNS expression of pro-inflammatory cytokines

**Fig. 6:**
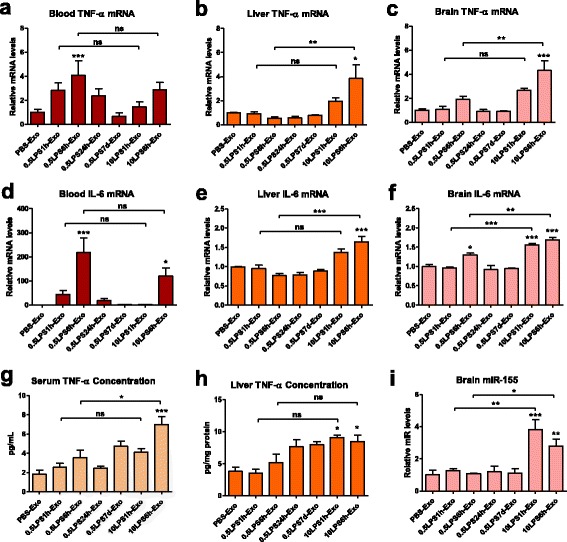
Exosomes isolated from LPS-treated mice increase the expression of pro-inflammatory cytokines in periphery and CNS. Whole blood, liver, and brain tissues were collected from the recipient mice 24 h after intravenous administration of 1 mg of exosomes (in 200 μl PBS) isolated from the donor mice which received one of the three LPS regimens. **a**–**f** The expression profile of TNF-α (**a**–**c**) and IL-6 (**d**–**f**) mRNA levels in the whole blood (**a** and **d**), liver (**b** and **e**), and brain (**c** and **f**) of the recipient mice. **g**, **h** TNF-α concentrations in the serum (**g**) and liver homogenates (**h**) from the recipient mice. **i** The levels of miR-155 in the brain of the recipient mice. *n* = 3–5 per group. Data represent mean ± SEM; ns no statistical significance, *P < 0.05, **P < 0.01, and ***P < 0.001

In order to determine whether the inflammatory effects of exosomes are tissue-specific or general, we collected whole blood and isolated liver and brain tissues from exosome-transfused mice for qRT-PCR analysis of pro-inflammatory cytokine expression. Interestingly, the exosomes isolated from LPS-treated mice increased TNF-α and IL-6 mRNA levels not only in the brain, but also in the blood and liver, suggesting that exosomes may be accepted at different locations throughout the body (Fig. 6a–f). Since we collected the samples 24 h after exosome injection, a time-point when Iba1 and GFAP signal peaked in the brain, we may have missed the peak time-points of peripheral TNF-α and IL-6 mRNA levels. To confirm the results, we measured TNF-α protein concentrations in sera and liver homogenates from mice treated with exosomes. TNF-α concentrations were elevated in both sera and liver by the treatment of high-dose-LPS-challenged exosomes (Fig. 6g, h). The TNF-α levels in all brain samples were scattered in the lower end of the detection range of the ELISA kit and showed no significant difference between groups, possibly because TNF-α levels in brain already returned to baseline after 24 h (data not shown). The expression of the pro-inflammatory microRNA miR-155 was increased by threefold in the brain by 10LPS6h-Exo and 10LPS24h-Exo, suggestive of neuroinflammation (Fig. 6i).

### Intraperitoneally administered LPS increases the expression of inflammation-related microRNAs in the serum-derived exosomes

**Fig. 7:**
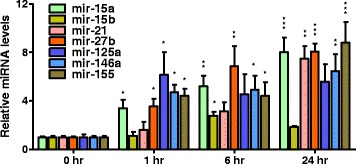
Increased expression of inflammatory microRNAs in the serum-derived exosomes from LPS-treated mice. The temporal profile of microRNA levels in the exosomes isolated from the sera of donor mice which received 0.5 mg/kg LPS. The microRNA levels were normalized to a synthetic spike-in small RNA control, *n* = 3–5 per group. Data represent mean ± SEM; *P < 0.05, **P < 0.01, and ***P < 0.001

Exosomes are lipid bilayer-enclosed extracellular vesicles containing microRNA, mRNA, protein, and lipid. We selected a panel of inflammation-related microRNAs from literature review and others’ reports. We found that many of these microRNA (miR-15a, miR-15b, miR-21, miR-27b, miR-125a, miR-146a, and miR-155) levels were significantly elevated in the serum-derived exosomes purified from mice starting from 1 h after i.p. injection of low-dose LPS and that many of the microRNA levels remained elevated after 24 h of LPS exposure (Fig. 7). In contrast, protein concentrations in these exosomes were not significantly altered during LPS treatment (Fig. 2b).

### Intravenously administered serum-derived exosomes are primarily taken up by microglial cells

**Fig. 8:**
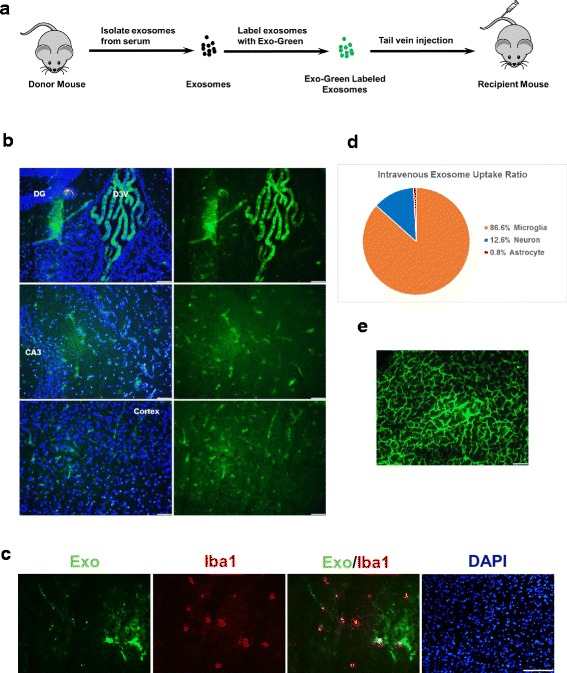
Uptake of intravenously administered exosomes in the brain. **a** A schematic diagram of the experimental procedure. Exosomes isolated from the sera of the donor mice were labeled with green fluorescence by the Exo-Green kit and injected intravenously to the recipient mice. **b** Lower magnification images of the brain sections in different areas. DG dentate gyrus, D3V dorsal third ventricle, CA3 field CA3 of hippocampus. DAPI was shown in blue. Scale bar, 50 μm. **c** Co-localization of the Exo-Green-labeled exosomes and microglia. The recipient mice were euthanized 24 h after the injection of Exo-Green-labeled exosomes (1 mg in 200 μl PBS). Brain sections were stained with anti-Iba1 for microglia (red) and with DAPI for nuclei (blue). Scale bar, 20 μm. **d** Quantification of the exosome uptake ratio by microglia, neuron, and astrocyte. **e** Representative image of the liver section showing uptake of exosomes labeled with green fluorescence. Scale bar, 50 μm

To determine whether exosomes can be directly taken up into the brain from the blood stream, we administered Exo-Green labeled intravenously into C57BL/6J mice. The experimental procedure was illustrated in Fig. 8a. Fresh snap-frozen mouse brains were sectioned to determine cellular uptake of exosomes using a fluorescent microscope. At both 6 and 24 h after the i.v. administration, we observed a small yet significant number of green fluorescent cells in the brain parenchyma. The 24-h time-point demonstrated a slight enhancement of green fluorescent cells but reduced fluorescent intensities, possibly due to decreased half-life of the Exo-Green dye in vivo (data not shown). Figure 8b is a representative image from the 24-h time-point. The fluorescently labeled exosomes were observed primarily in the ependymal cells in the third and lateral ventricles, suggesting that these areas may be the main entry sites via blood cerebral spinal fluid brain barrier (BCSFB) for the exosomes to translocate into brain parenchyma (Fig. 8c). Staining of the brain slices with Iba1 antibody revealed that the majority cell type of the green fluorescent cells was microglia, accounting for ~ 86.6% (Fig. 8d). Sections from the liver demonstrated robust uptake of labeled exosomes in the liver (Fig. 8e).

### Potentially direct causative role of peripheral LPS-exosomes in the induction of neuroinflammation, microglial activation in particular

**Fig. 9:**
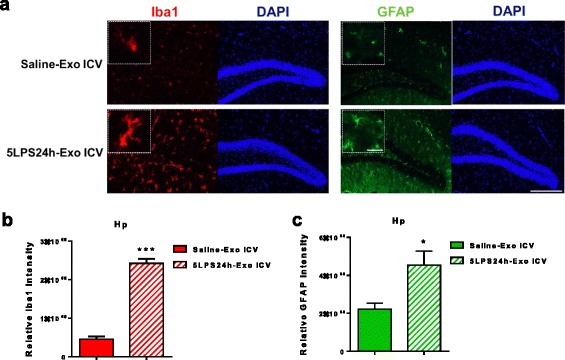
I.c.v. infusion of peripheral LPS-exosomes provoked dramatic microglial activation and moderate astrocytic activation. **a** Representative photomicrographs of the immunofluorescent staining in the brain sections from Saline-Exo ICV- and 5LPS24h-Exo ICV-treated mice. Anti-Iba1 antibody for microglia (red) and anti-GFAP antibodies for astrocytes (green) and DAPI for nuclei (blue). Scale bar, 50 μm. The white-dotted boxes are zoom-in views of the corresponding photomicrographs, scale bar 5 μm. **b**, **c** Quantification of the relative immunofluorescent intensity of Iba1 (**b**) and GFAP (**c**). *n* = 3 per group. Data represent mean ± SEM; *P < 0.05 and ***P < 0.001

To provide further evidence for a direct causative role of peripheral exosomes to CNS inflammation, we injected normal mice with serum-derived exosomes from LPS-challenged donor mice and determined neuroinflammation 24 h later. As shown earlier in Fig. 2d, the serum-derived exosomes purified from mice receiving LPS for 24 h contained undetectable endotoxin. The i.c.v. infused exosomes isolated from LPS-challenged donor mice induced markedly increased neuroinflammation (Fig. 9b, c) with most prominent effect in microglia. Although GFAP is widely accepted to be the best marker for both resting and activated astrocytes which can be distinguished by their morphologies, for some unknown reasons, GFAP only stain astrocytes in hippocampi but not much in cortical regions, arguing against astrocytes being as one homogenous population. Additional marker S100 staining revealed similar results and a vast majority of S100-positive cells matched to the GFAP-positive cells, though at distinct cellular compartments (Additional file 4: Figure S4).

## Discussion

In this study, we investigated a specific mechanism of peripheral contribution to neuroinflammation. Using a LPS model, we show that the microglial cells and astrocytes in mouse brain are activated in a dose- and time-dependent manner by peripherally administered LPS. This observation is consistent with many other reports [33]. A higher dose LPS (> 3 mg/kg) challenge has been known to induce blood-brain barrier (BBB) disruption, followed by leukocyte recruitment in the brain and CNS inflammatory response [34–36]. While most research in general utilize doses between 0.5–10 mg/kg, a dose of LPS as low as 0.1 mg/kg administered intraperitoneally is sufficient to induce microglial activation in mice without compromising the BBB components [37]. Using different systemic LPS treatment regimens: single low- (0.5 mg/kg) and single high (10 mg/kg) dose, as well as multiple injections (0.5 mg/kg daily for 7 days), we compared the effect of dose, treatment time, and multiple challenges on glial activation. LPS can activate the CNS immune pathways and processes independently of BBB disruption [36]. A similar conundrum has been present in the field of AD [38]. Efforts to unravel this mysterious tangle have been substantially undermined in part due to the historical concept of “CNS immune privilege.” Here, we present novel findings that the serum-derived exosomes, when transfused from LPS-challenged mice into C57BL/6J mice via tail-vein enhance microglial and astrocytic activation and increase the expression of inflammatory cytokines in the brain. In addition, we compared the effects of serum-derived exosomes from LPS-challenged donor mice in recipient mice through i.v. and i.c.v. routes. Of note, the i.c.v. infused LPS exosomes induced comparable level of microglial activation in the hippocampal regions with i.v. injection (Figs. 5c and 9b, c and Additional file 2: Figure S2). Our observations suggest that exosomes may be one factor mediating the activation of neuroinflammatory process during systemic peripheral inflammation. Our results may also shed light on mechanisms of communication between the brain immune surveillance and the peripheral immune system.

Exosomes are taken up by the acceptor cells mainly through endocytosis and membrane fusing [39]. It has been reported that the blood-borne exosomes are internalized by dendritic cells or macrophages through phagocytosis, a specific form of endocytosis [24]. Using the fluorescently labeled exosomes, we are able to observe an accumulation of green fluorescence in brain cells 6–24 h after intravenous injection, and mainly in the microglia. However, the number of cells with noticeable fluorescent signal in brain parenchyma was surprisingly low; the green fluorescent intensity was strongest in the areas around the third and the lateral ventricles, especially in the ependymal cells. Our findings are consistent with several reports on exosome biodistribution, in which the majority of fluorescently labeled or radiolabeled exosomes injected intravenously are accumulated in the liver, spleen, and lung within less than 1 h, while barely detectable in brain [40–43]. In one of the reports, using an in vivo nanoparticle imaging system, the authors show that brain accumulates detectable level of fluorescently labeled exosomes around the third ventricle and lateral ventricle areas at 24 h after intravenous injection, but to a much lesser extent in comparison with that in the liver, spleen, and lung [42]. Considering serum-derived exosomes are heterogeneous in size and source, exosomes derived from different cell types may have distinct tissue-specific homing effects [44]. The observed low efficiency of brain exosome uptake may be attributed to a small population of exosome subtypes which preferentially target to the brain in the variety of serum-derived exosomes. Moreover, the prominent microglial uptake may also imply a predominant source of donor exosomes secreted from peripheral monocyte-macrophage-lineage cells, which would be consistent with the common knowledge that LPS response is mediated by the myeloid cells. While the level of astrocytic exosome uptake was low, the observation of astrocytic activation by exosome infusion is clear. We speculate that the astrocytic activation was a secondary event of the activated microglia, as recently reported [45]. It should be noted that the fluorescent or radioactive tracer, which is a lipophilic or membrane permeable chemical, may also alter the nature and tissue distribution of exosomes. A more suitable and reliable/stable labeling strategy is under development to further investigate the exosome homing cell types and mechanisms. Another possible explanation for the low percentage of green fluorescence incorporated neural cells after the i.v. injection of labeled exosomes might be that the peripheral exosomes need to be first packaged by a certain cell type such as epithelial cells or ependymal cells and released again before they can be taken up by the neural cells. Although we cannot rule out possible involvement of other intermediate cell types such as the epithelium lining up the blood CSF brain barrier to be the major sites for the uptake and repackage of peripheral exosomal contents, the observation that i.c.v. infused LPS exosomes induce similar degrees of neuroinflammation implies a direct causative role of peripheral exosomes.

In addition to the LPS model, we also employed the HFD-induced obesity model. HFD has long been known to induce gliosis in the hypothalamus [18, 29–32]. Gliosis was increased as early as 1 day after the feeding, when there was no leukocyte recruitment to the brain [46]. The effect of HFD on microglial activation in the hippocampus areas has also been reported after chronic feeding [19]. In line with these previous studies, we showed that HFD feeding for 16 weeks in C57BL/6J mice increased microglial activation in the hippocampus and neocortex. Similarly, the exosomes purified from HFD-fed sera acutely increased the microglial response in the two brain regions after 24 h, providing a proof-of-concept that exosomes mediate the neuroinflammation in HFD-induced obesity. One limitation of the study was that the amounts of exosomes applied may overestimate the pathophysiological amounts. Considering that development of neuroinflammation during HFD feeding is a chronic long-term process, one can infer that serum exosome uptake by the brain cells is also a continuous process along the feeding period. It is possible that the brain accepts negligible or minimal amounts of serum-derived exosomes on a daily basis. Future work will be needed to examine the effect of a long-term treatment regimen (e.g., repeated injections) with low-dose exosomes in order to more closely approximate the physiological conditions.

It is also unclear what the sources of these brain-targeting serum-derived exosomes are, as exosomes can be secreted by almost all types of cells. Identifying the sources is a challenging feat, but not an impossible one. Cell type specific markers either on the exosome membrane or within the exosomes can be used as a way to identify the host cells; however, current research in this field is primitive [47]. The observations that microglia being the major subtype of glial cells affected by LPS-exosomes strongly suggest that the effective exosomes are from a myeloid source, consistent with the notion that mononuclear and dendritic cells are the major mediators for LPS-induced systemic inflammation.

In our study, exosome abundance in the sera was not affected by systemic inflammation, as their protein concentrations in serum-derived exosomes were unaltered, suggesting that it was the identity changes in the contents (i.e., mRNA, microRNA, protein, or lipid) in the LPS-treated exosomes that caused systemic and CNS inflammation. Our study pioneered research that characterizes detailed time-course response of the circulating exosomal microRNAs under LPS challenge. We show that the serum-derived exosomes purified from mice with LPS challenge (< 1 h) contained elevated expression of inflammation-related microRNAs, including the miR-21, miR-125a, miR-146a, and miR-155, all of which have been involved in regulating Toll-like receptor (TLR) signaling [48].

## Conclusions

In conclusion, we have provided experimental in vivo evidence that the purified serum-derived exosomes from LPS-treated mice are devoid of LPS contamination and can induce systemic and CNS inflammation, implying that peripheral circulating exosomes contribute to neuroinflammation in conditions with systemic inflammation. Increased expression of inflammatory microRNAs in serum-derived exosomes during the process of systemic inflammation may play a role in regulating the systemic and CNS immune response. Although the direct causative role of the circulating exosomes in CNS inflammation via migration into brain was compromised by systemic inflammation and low number of exosomal uptake events in the brain, i.c.v. infusion of purified circulating inflammatory exosomes induced dramatic microgliosis and moderate astrogliosis. Further research on how the peripheral serum-derived exosomes cross the BBB to reach the recipient brain parenchymal cells would be worth pursuing to reveal the detailed mechanisms involved in both increased neuroinflammation and exosomal uptake by the neural cells.

## Additional files

Additional file 1: Figure S1. Increased cell number of microglia and astrocytes after intraperitoneally injected LPS. **a** and **b** Number of Iba1+ (**a**) and GFAP+ (**b**) cells in the hippocampal area between different treatments. **c** Number of the Iba1+ cells in the neocortical area between treatments. *n = 5-8* per group. Data represent mean ± SEM; ns, no statistical significance, *P < 0.05, **P < 0.01, and ***P < 0.001. (PDF 18 kb)

Additional file 2: Figure S2. The donor mice received either saline or 5 mg/kg of LPS injection and were kept alive for 24 h before blood collection. The recipient mice were iv. injected with the purified exosomes and kept alive for 24 h before being sacrificed. **a** Quantification of the relative immunofluorescent intensity of Iba1, GFAP, and S100 in the cortical area in Saline-Exo- and 5LPS24h-Exo-treated mouse brains. *n = 3* per group. **b** Representative photomicrographs of the immunofluorescent staining in the brain sections from Saline-Exo- and 5LPS24h-Exo-treated mice. Anti-Iba1 antibody for microglia and anti-S100 antibodies for astrocytes (*red*), and DAPI for nuclei (*blue*). Scale bar: 50 μm. The white dotted boxes are zoom-in views of the corresponding photomicrographs, scale bar 5 μm. Data represent mean ± SEM; *P < 0.05. (PDF 87 kb)

Additional file 3: Figure S3. Serum-derived exosomes isolated from HFD-fed mice increase microglial activation. **a** Photomicrographs of the hippocampal (Hp) and neocortical (Cx) areas from mice fed with either a chow diet or a HFD for 16 weeks. Brain sections were stained with anti-Iba1 antibody for microglia (*red*) and with DAPI for nuclei (*blue*). Scale bar: 50 μm. The white dotted boxes are zoom-in views of the corresponding photomicrographs, scale bar 10 μm. **b** and **c** Comparison of the relative immunofluorescent intensity of Iba1 in the hippocampal (**b**) and neocortical (**c**) area between two groups. *n = 4-6* per group. **d** Average body weight of Chow-fed and HFD-fed mice before and after 16 weeks of feeding. *n* = *10* per group. **e** Photomicrographs of the hippocampal and neocortical areas from the recipient mice. Exosomes (1 mg in 200 μl PBS) isolated from the sera of Chow-fed or HFD-fed mice were injected intravenously to C57BL/6J mice. The recipient mice were euthanized 24 h after the injection. Brain sections were stained with anti-Iba1 antibody for microglia (*red*) and with DAPI for nuclei (*blue*). Scale bar: 20 μm. **f** and **g** Comparison of the relative immunofluorescent intensity of Iba1 in the hippocampal (**f**) and neocortical (**g**) area between two groups. *n = 5* per group. Data represent mean ± SEM; *P < 0.05, ***P < 0.001. (PDF 178 kb)

Additional file 4: Figure S4. GFAP and S100 antibodies recognize the same cells in the hippocampus. Brain sections were stained with anti-GFAP antibody (*green*), with anti-S100 antibody (*red*), and with DAPI for nuclei (*blue*). Scale bar: 25 μm. (PDF 88 kb)

## Abbreviations

AD: Alzheimer’s disease
ALS: Amyotrophic lateral sclerosis
BBB: Blood-brain barrier
BCA: Bicinchoninic acid
CD68: Cluster of differentiation 68
CNS: Central nervous system
ELISA: Enzyme-linked immunosorbent assay
FTD: Frontotemporal lobar dementia
GFAP: Glial fibrillary acidic protein
GWAS: Genome-wide association studies
HFD: High-fat diet
i.p.: Intraperitoneal
Iba1: Ionized calcium-binding adaptor molecule 1
IL-6: Interleukin 6
LPS: Lipopolysaccharide
NSAIDs: Nonsteroidal anti-inflammatory drugs
OCT: Optimal cutting temperature
PBS: Phosphate-buffered saline
PD: Parkinson’s disease
qRT-PCR: Quantitative real-time polymerase chain reaction
TLR: Toll-like receptor
TNF-α: Tumor necrosis factor alpha
TSG101: Tumor susceptibility gene 101 protein
