# Supplementary figures and images for: In vivo evidence for the contribution of peripheral circulating inflammatory exosomes to neuroinflammation
Source: J Neuroinflammation. 2018 Jan 8;15:8. doi: 10.1186/s12974-017-1038-8 (PMC5759808; doi:10.1186/s12974-017-1038-8)

Supplementary Figure 1.

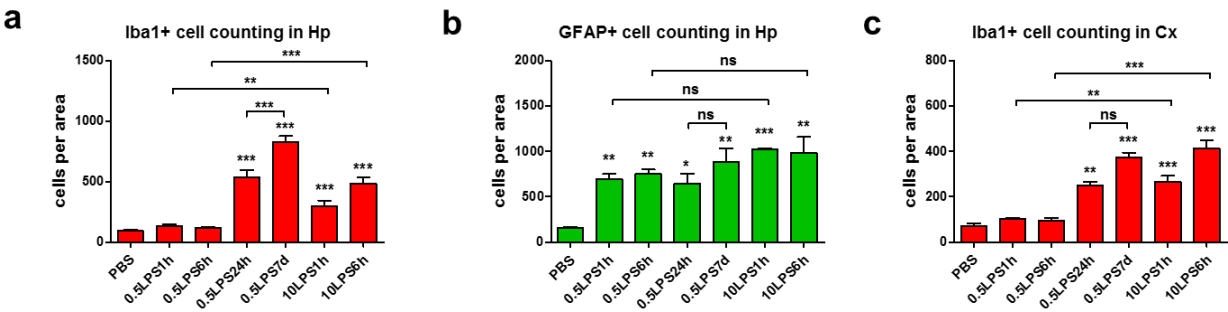

Supplement: Supplementary file 1 — Increased cell number of microglia and astrocytes after intraperitoneally injected LPS. a and b Number of Iba1+ (a) and GFAP+ (b) cells in the hippocampal area between different treatments. c Number of the Iba1+ cells in the neocortical area between treatments. n = 5-8 per group. Data represent mean ± SEM; ns, no statistical significance, *P < 0.05, **P < 0.01, and ***P < 0.001. (PDF 18 kb) [file 12974_2017_1038_MOESM1_ESM.pdf]

Supplementary Figure 2.

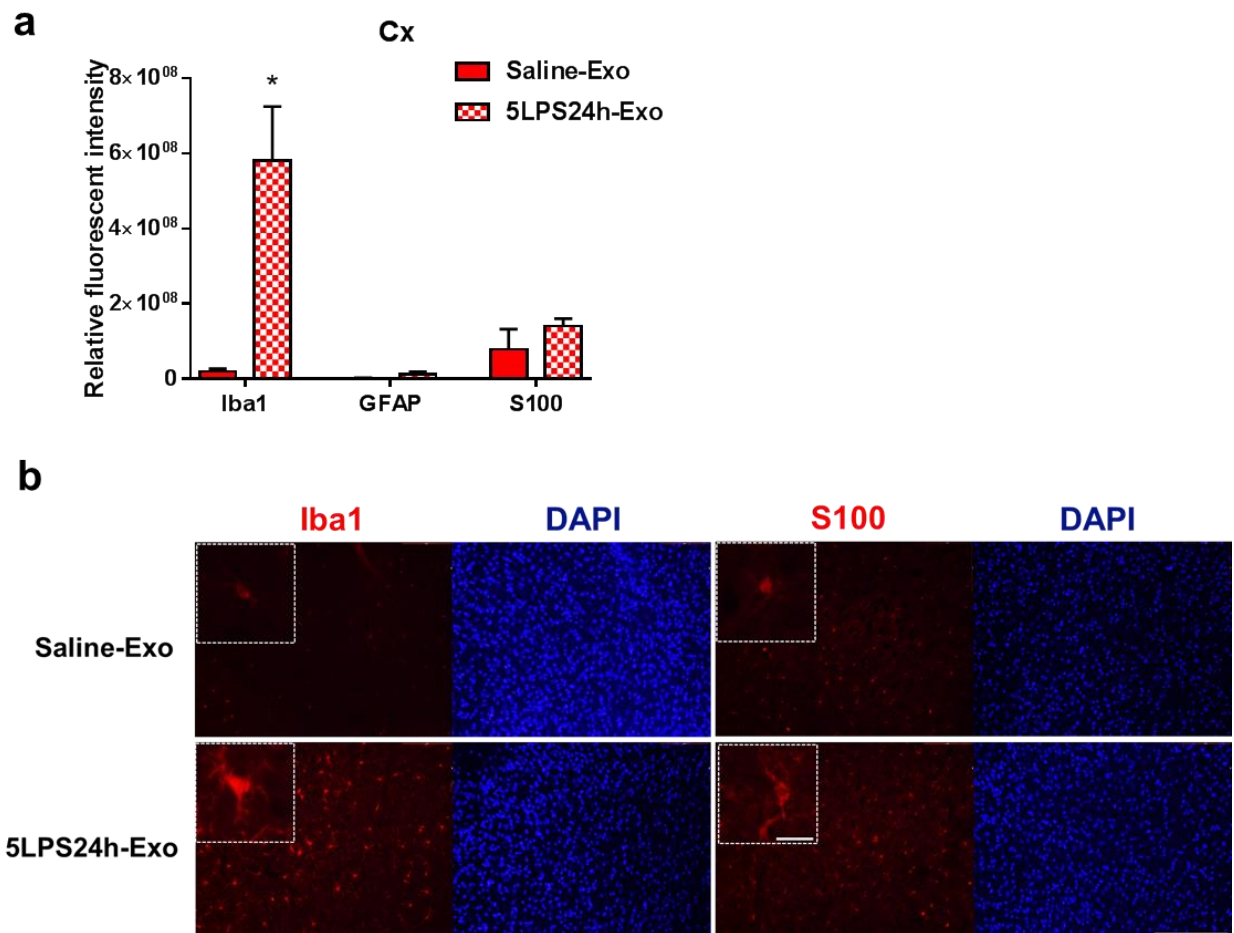

Supplement: Supplementary file 2 — The donor mice received either saline or 5 mg/kg of LPS injection and were kept alive for 24 h before blood collection. The recipient mice were iv. injected with the purified exosomes and kept alive for 24 h before being sacrificed. a Quantification of the relative immunofluorescent intensity of Iba1, GFAP, and S100 in the cortical area in Saline-Exo- and 5LPS24h-Exo-treated mouse brains. n = 3 per group. b Representative photomicrographs of the immunofluorescent staining in the brain sections from Saline-Exo- and 5LPS24h-Exo-treated mice. Anti-Iba1 antibody for microglia and anti-S100 antibodies for astrocytes (red), and DAPI for nuclei (blue). Scale bar: 50 μm. The white dotted boxes are zoom-in views of the corresponding photomicrographs, scale bar 5 μm. Data represent mean ± SEM; *P < 0.05. (PDF 87 kb) [file 12974_2017_1038_MOESM2_ESM.pdf]

Supplementary Figure 3.

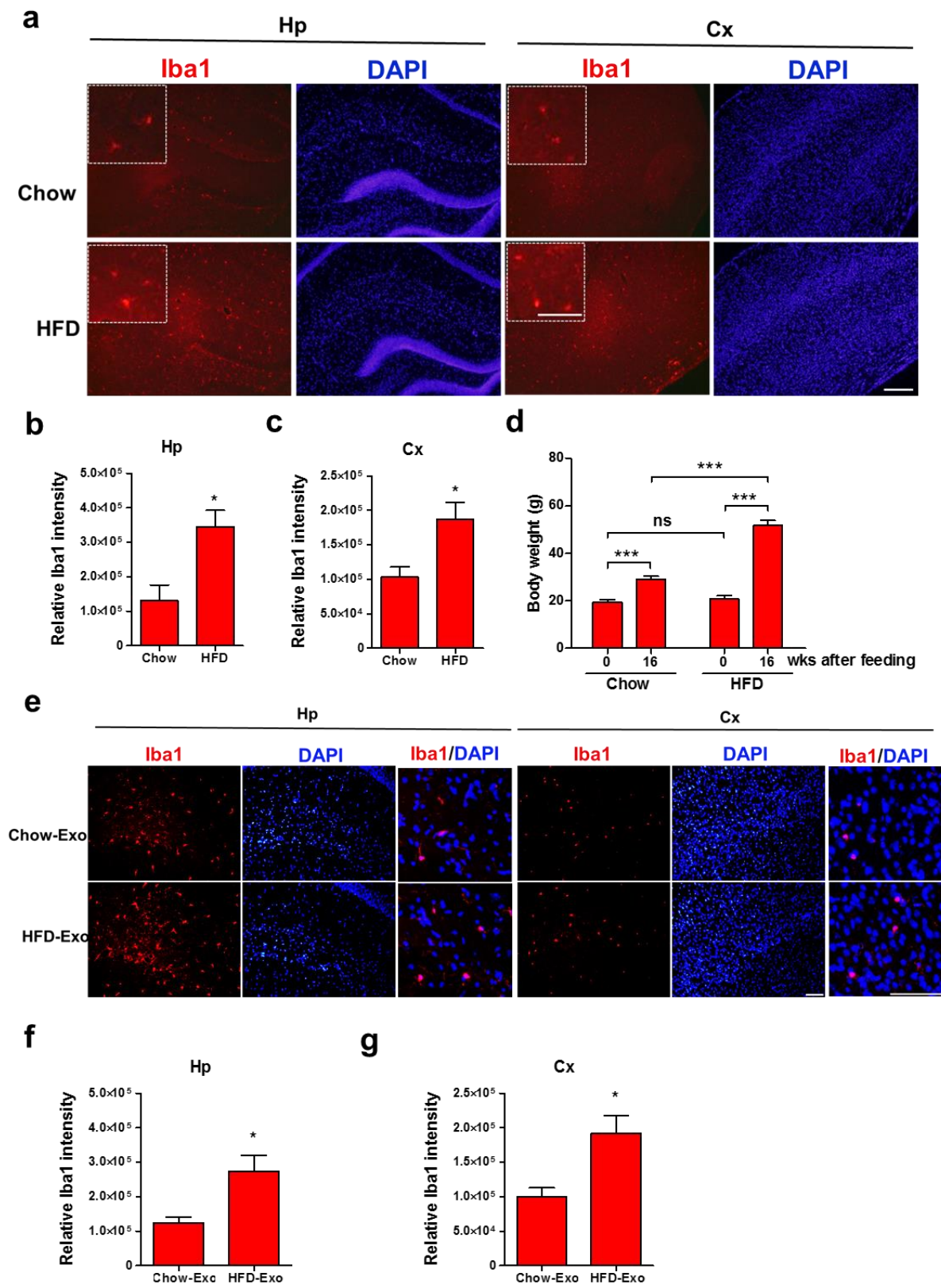

Supplement: Supplementary file 3 — Serum-derived exosomes isolated from HFD-fed mice increase microglial activation. a Photomicrographs of the hippocampal (Hp) and neocortical (Cx) areas from mice fed with either a chow diet or a HFD for 16 weeks. Brain sections were stained with anti-Iba1 antibody for microglia (red) and with DAPI for nuclei (blue). Scale bar: 50 μm. The white dotted boxes are zoom-in views of the corresponding photomicrographs, scale bar 10 μm. b and c Comparison of the relative immunofluorescent intensity of Iba1 in the hippocampal (b) and neocortical (c) area between two groups. n = 4-6 per group. d Average body weight of Chow-fed and HFD-fed mice before and after 16 weeks of feeding. n = 10 per group. e Photomicrographs of the hippocampal and neocortical areas from the recipient mice. Exosomes (1 mg in 200 μl PBS) isolated from the sera of Chow-fed or HFD-fed mice were injected intravenously to C57BL/6J mice. The recipient mice were euthanized 24 h after the injection. Brain sections were stained with anti-Iba1 antibody for microglia (red) and with DAPI for nuclei (blue). Scale bar: 20 μm. f and g Comparison of the relative immunofluorescent intensity of Iba1 in the hippocampal (f) and neocortical (g) area between two groups. n = 5 per group. Data represent mean ± SEM; *P < 0.05, ***P < 0.001. (PDF 178 kb) [file 12974_2017_1038_MOESM3_ESM.pdf]

Supplementary Figure 4.

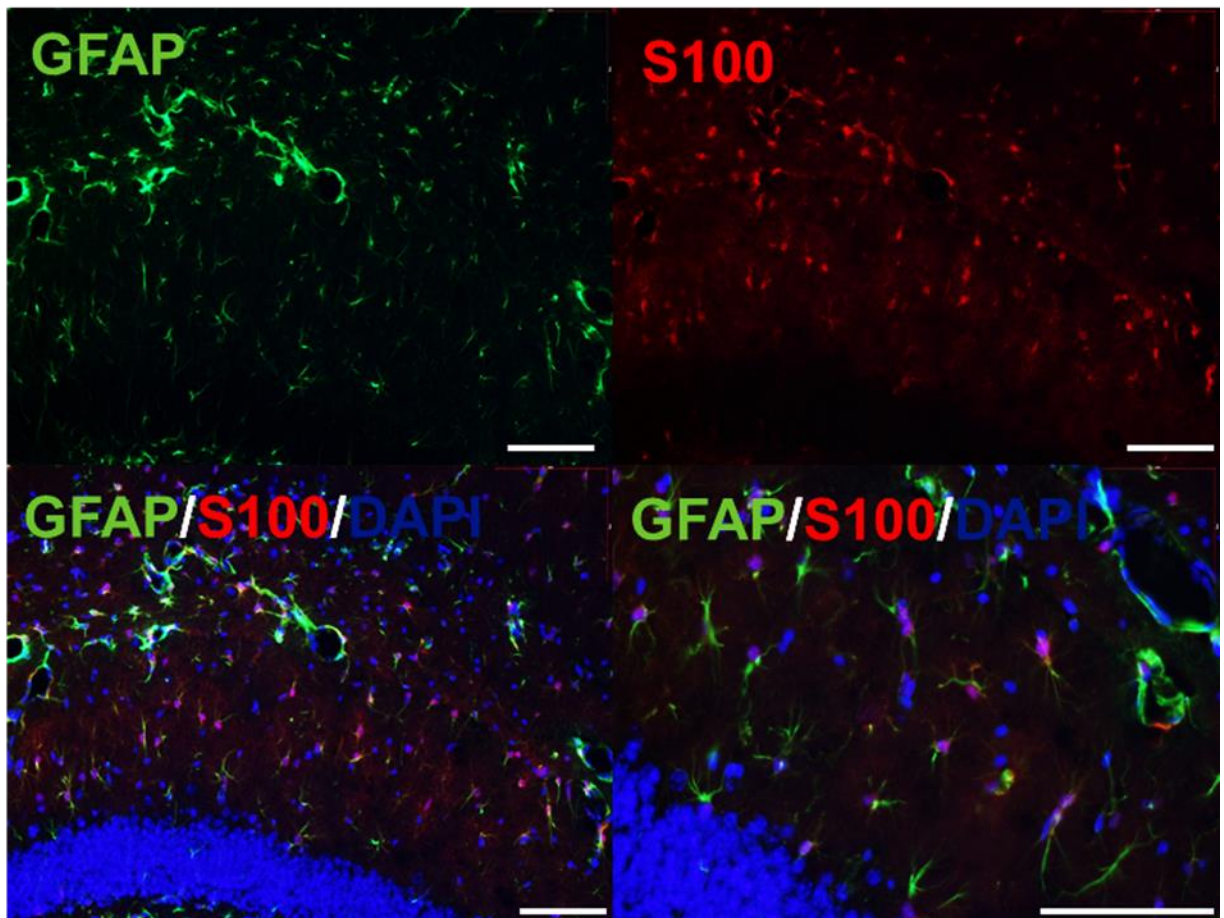

Supplement: Supplementary file 4 — GFAP and S100 antibodies recognize the same cells in the hippocampus. Brain sections were stained with anti-GFAP antibody (green), with anti-S100 antibody (red), and with DAPI for nuclei (blue). Scale bar: 25 μm. (PDF 88 kb) [file 12974_2017_1038_MOESM4_ESM.pdf]
